# The path to next generation biofuels: successes and challenges in the era of synthetic biology

**DOI:** 10.1186/1475-2859-9-3

**Published:** 2010-01-20

**Authors:** Clementina Dellomonaco, Fabio Fava, Ramon Gonzalez

**Affiliations:** 1 Department of Chemical and Biomolecular Engineering, Rice University, Houston, TX, USA; 2 Department of Applied Chemistry and Material Science, University of Bologna, Italy; 3 Department of Bioengineering, Rice University, Houston, TX, USA

## Abstract

Volatility of oil prices along with major concerns about climate change, oil supply security and depleting reserves have sparked renewed interest in the production of fuels from renewable resources. Recent advances in synthetic biology provide new tools for metabolic engineers to direct their strategies and construct optimal biocatalysts for the sustainable production of biofuels. Metabolic engineering and synthetic biology efforts entailing the engineering of native and *de novo *pathways for conversion of biomass constituents to short-chain alcohols and advanced biofuels are herewith reviewed. In the foreseeable future, formal integration of functional genomics and systems biology with synthetic biology and metabolic engineering will undoubtedly support the discovery, characterization, and engineering of new metabolic routes and more efficient microbial systems for the production of biofuels.

## Introduction

The increased use of fossil fuels has caused greenhouse gas emissions and created undesirable damage to the environment. Current instability of oil supplies and the continuous fluctuation of prices have further ignited widespread interest in alternative energy sources. These factors, which revolve around economical, environmental, and geopolitical issues, are central to current interest in renewable energy sources [1].

An entire branch of biotechnology, referred to as "white biotechnology"[2], embraces the bioproduction of fuels and chemicals from renewable sources. These technologies use living cells and enzymes to synthesize products that are easily (bio)degradable, require less energy and create less waste during their production or use than those produced from fossil resources.

While the concept of biofuels was conceived in the 1970s when the world faced a large-scale oil crisis, recent advances in synthetic biology [3,4], metabolic engineering [4-7], and systems biology [8,9] have generated a renewed interest in the production of biofuels. Microbial factories for the synthesis of biofuels and amenable to industrial applications are being constructed by assembling natural and *de novo *pathways that re-direct carbon to the desired products [10-16]. Gene expression is modulated to fine-tune microbial metabolism for optimal production and proteins engineered to acquire new catalytic activities or to improve native properties [17-19]. "*omics*" technologies have been developed to analyze and model systems in a holistic manner and address complex questions about the functioning of native and synthetic networks in microbial cells [20]. New sequencing technologies (NST) enabling quick identification and analysis of genomic variations such as single nucleotide polymorphisms (SNPs), copy number variations (CNVs), translocations, and insertions and deletions [21,22], are being instrumental to understand complex microbial environments, uncover diversity and characterize the genetic makeup of various species of microorganisms [23] that could hold promise for generating biofuels. Continuing efforts in the last decades in the field of metabolic engineering have paved the way to the engineering of efficient synthetic pathways for the production of biofuels [4,6,7,11].

**Figure 1:**
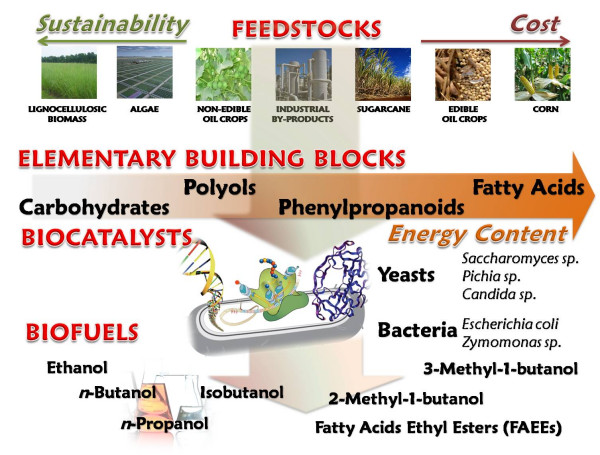
**General bioprocess scheme for the production of fuels from renewable feedstocks**. Different feedstocks are listed according to their environmental and economical sustainability. Feedstock deconstruction releases elementary building blocks such as pentoses, hexoses, polyols, fatty acids, etc. that are then microbially converted to biofuels.

Because of its abundance and renewable nature, biomass has the potential to offer diverse supplies of reliable, affordable, and environmentally sound biofuels to replace fossil fuels. Given the complexity of biomass in terms of chemical composition, a conventional bioprocess for fuels production entails several steps such as collection of biomass, feedstock deconstruction to obtain biomass constituents (e.g., monosaccharides, fatty acids, etc.) and their conversion to biofuels (Fig. 1).

This review will focus on the role of metabolic engineering and synthetic biology as enabling technologies for the production of alcohol biofuels (i.e. ethanol and butanol). Although great efforts have been devoted to development of gaseous biofuels as well as the exploitation of the side-streams generated by the utilization of biomass for biofuels production (biorefinery concept), these topics are beyond the scope of this review and have been discussed elsewhere [24-26].

### Renewable feedstocks for biofuels production

Many biomass feedstocks can be used for the production of biofuels (Fig. 1). These include agricultural lignocellulosic residues, edible and non-edible crops, and waste streams (e.g. bagasse from sugar manufacture, industrial by-products) (Fig. 1). Lignocellulosic biomass varies among species but generally consists of ~25% lignin and ~75% carbohydrate polymers (cellulose and hemicellulose) [27] and it is the largest known renewable carbohydrate source. Oil seed crops, on the other hand, are mainly composed of various triacylglycerols (TAGs), molecules consisting of three fatty acids chains (usually 18- or 16-C long) esterified to glycerol [28].

Starch (i.e. corn, wheat, barley, etc.) and sugar crops (i.e. cane, beet, etc.) are the primary feedstocks currently used for bioconversion to ethanol, while TAGs extracted from oil seed crops (i.e. soybean, oil palm, sunflower, etc.) are chemically esterified to biodiesel. Both of these processes are presently under debate since they employ edible feedstocks. As shown in Fig. 1, they are expensive and non-sustainable feedstocks that might adversely impact the food-feed chain. Lignocellulosic feedstocks can be converted into fuels either thermochemically or biologically (Fig. 1). Major challenges for biological conversion are posed by biomass recalcitrance. The cellulosic and hemicellulosic portions of biomass can be separated from the lignin and depolymerized by enzymatic hydrolysis to obtain the constituent sugars, mainly glucose, xylose, and arabinose (Fig. 1) [27]. Conversely, since lignin serves as a structural material, it prevents access of hydrolytic enzymes, hindering its biological conversion. To overcome its recalcitrance, feedstock deconstruction is therefore required [28]. Processing routes for oil seed crops instead entails pressing or solvent/supercritical extraction of triacylglycerols [29,30].

In contrast, utilization of lignocellulosic or non-edible oil seed crops is sustainable and renewable [27]. For example, many algal species have been found to grow rapidly and produce substantial amounts of triacylglycerols or oil (oleaginous algae). Therefore, it is forecasted that algae could be employed as cell factories to produce biofuels [31,32]. Algae offer many advantages in the search for sustainable, renewable bioenergy feedstocks and have the potential to provide orders of magnitude more oil per acre of land than traditional oil seed crops [33]. Further, algae can be grown in arid climates with brackish water or seawater and use carbon dioxide as nutrient. Open ponds will likely be the only cost and energy effective means of production for algal biofuels feedstocks for the foreseeable future [34]. There are, though, major disadvantages associated with the use of open pond systems; they require highly controlled environments due to inherent threat of microbial contamination and yield low biomass concentration in the microalgal culture due to the limit of light penetration [34]. Nevertheless, these problems are expected to be overcome or minimized by technology development/improvement.

Vast supplies of diverse renewable resources are therefore available for conversion to generic feedstock constituents (carbohydrates, polyols, fatty acids, etc.) that can be microbially converted into valuable fuels (Fig. 1).

### Synthesis of biofuels from carbohydrate-based feedstocks

Six-carbon (6-C, hexoses) and five-carbon (5-C, pentoses) sugars are the most abundant biomass constituents. Several metabolic engineering and synthetic biology strategies have been implemented in the past decades to convert them, individually or as a sugar mixture, to different biofuels.

#### Conversion of sugars to ethanol

**Figure 2:**
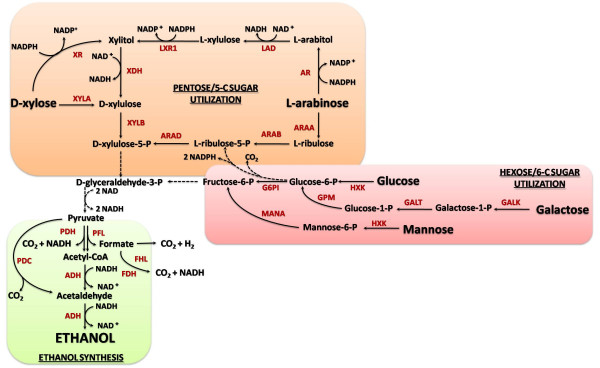
**Engineered pathways for microbial production of ethanol from carbohydrates**. Orange, red and green boxes indicate pathways for pentose and hexose sugars utilization, and ethanol synthesis respectively. The dashed lines indicate multiple steps. Abbreviations: ADH, alcohol dehydrogenase; AR, aldose reductase; ARAA, L-arabinose isomerase; ARAB, L-ribulokinase; ARAD, L-ribulosephosphate 4-epimerase; FDH, formate dehydrogenase; FHL, formate hydrogen lyase; LAD, L-arabitol 4-dehydrogenase; LXR1, L-xylulose reductase; PDC, pyruvate decarboxylase; PDH, pyruvate dehydrogenase; PFL, pyruvate formate lyase; XDH, xylitol dehydrogenase; XR, xylose reductase; XYLA, xylose isomerase; XYLB, xylulokinase.

Baker's yeast (*Saccharomyces cerevisiae*) has long been used in the brewery industry to produce ethanol from 6-C sugars (Fig. 2) but this organism is unable to ferment 5-C sugars. Many bacteria, on the other hand, produce ethanol as a natural product of hexose fermentation, but this biofuel represents only a small fraction of the product mixture (mixed-acid fermentation) [35].

Synthetic biology and metabolic engineering have been extensively used in *S. cerevisiae*, *Zymomonas mobilis *and *Escherichia coli *to enhance ethanol fermentation (Fig. 2) [27,35-38]. Many microorganisms, including bacteria and yeasts, can produce ethanol as the main fermentation product from carbohydrates [39]. Since neither *S. cerevisiae *nor *Z. mobilis*, currently used to carry out industrial ethanol fermentation, can use xylose or arabinose (the most abundant lignocellulosic sugars, next to glucose), microorganisms other than *S. cerevisiae *have come to the forefront in bioethanol production from lignocelluosic biomass. Indeed, many microorganisms are able to efficiently utilize pentose sugars but cannot naturally produce ethanol at sufficient yield and productivity. Some microorganisms that utilize pentoses, such as the bacteria *E. coli *and *Klebsiella oxytoca *and the yeast *Pichia stipitis*, have been successfully engineered for ethanol production [36]. Alternatively, pentose catabolic pathways have been expressed in ethanologenic microorganisms, such as the conventional yeast *S. cerevisiae *[40,41] or the ethanologenic bacterium *Z. mobilis *[42]. These efforts are discussed in detail below.

Two natural enzymatic pathways for xylose consumption are known to exist (Fig. 2), and both have been independently transferred to *S. cerevisiae*. In one pathway the conversion of D-xylose to D-xylulose is performed by a xylose isomerase (XI). Since yeasts can grow on and ferment xylulose, a heterologous bacterial xylose isomerase (XI) was expressed in *S. cerevisiae *for xylose catabolism. However, all early efforts using this approach failed despite successful cloning and expression of the gene *xylA *from *Thermus thermophilus *[43,44] and *Piromyces *sp E2 [44] which produced an active XI in *S. cerevisiae*. The failure was partially because xylose isomerase is strongly inhibited by xylitol, favoring isomerization equilibrium toward xylose formation. More recently, a genetically engineered strain expressing the heterologous *xylA *gene from the anaerobic fungus *Piromyces *sp E2a was evolved to anaerobically grow on xylose [38,44] and produced high yield of ethanol (0.42 g/g xylose). This approach demonstrated that the *S. cerevisiae *metabolic pathway could be better engineered through a combination of rational and combinatorial approaches.

The second natural pathway primarily found in certain fungi and yeast species consists of two enzymatic steps: aldose (xylose) reductase (XR) and xylitol dehydrogenase (XDH) (Fig. 2). Approaches that have used this pathway for engineering *S. cerevisiae *rely on the corresponding genes (*xyl*1 and *xyl*2) from the xylose-fermenting yeast *P. stipitis *[45,46]. The introduction of either pathway enables *S. cerevisiae *to consume xylose [47]. However, this strategy was not successful, because the recombinant strain, which converts xylose to xylulose by the combined action of NADPH-dependent xylose reductase and NAD-linked xylitol dehydrogenase, cannot sustain its anaerobic growth due to an imbalance of reducing equivalents (i.e., NADH accumulation and NADPH depletion), which also results in the excretion of xylitol [48]. This is because the NADH generated by the xylitol dehydrogenase reaction cannot be used to produce NADPH for xylose reduction due to the lack of a transhydrogenase that interconverts NADPH and NADH [49]. Although the reducing equivalents in excess could be effectively removed via aeration, this would shift cell metabolism from fermentation to respiration and limit ethanol production. Various approaches to alleviate the cofactor imbalance have been reported, including the control of XR/XDH expression ratio to a low value [46], mutations to reduce the affinity of XR for NADPH [50] and XDH for NAD^+ ^[51], and shifting the cofactor specificity of XDH from NAD^+ ^to NADP^+ ^[51]. The ammonium assimilation pathway mediated by two glutamate dehydrogenases was altered by deleting GDH1 (NADPH-dependent) and overexpressing GDH2 (NADH-dependent), resulting in 44% reduction in xylitol accumulation and 16% increase in ethanol yield [52]. Metabolic flux analysis using ^13^C labeling showed that the modification had shifted the cofactor preference of XR from NADPH to NADH [53]. Z. *mobilis*, another ethanologenic bacterium, is able to produce high titers of ethanol from glucose and sucrose, but not pentoses. To introduce xylose metabolism, *E. coli *genes encoding for xylose isomerase, xylulokinase, transketolase, and transaldolase were expressed in *Z. mobilis *CP4 (pZB5), allowing for growth on xylose with 86% ethanol yield [42]. Similarly, arabinose metabolism was introduced in *Z. mobilis *ATCC39676 (pZB206) by expression of *E. coli *genes *araABD *(L-arabinose isomerase, L-ribulokinase, L-ribulose-5-P-4-epimerase), as well as genes encoding for transketolase and transaldolase, resulting in growth on arabinose with 98% ethanol yield [54]. In both cases, xylose or arabinose are first converted to xylulose-5-P, and then proceed into the pentose phosphate pathway to yield glyceraldehyde-3-P, which is an intermediate in the Embden-Meyerhof-Parnas (EMP) pathway (Fig. 2). The recombinant *Z. mobilis *can produce ethanol from pentoses only at low concentrations, limiting its potential for industrial applications [55].

An alternative to developing a pentose-fermenting ethanologenic strain is to construct synthetic pathways for ethanol production in hosts that can utilize pentoses. Wild-type *E. coli *has an excellent range of substrate utilization, including all the lignocellulosic sugars (glucose, xylose, arabinose, mannose, galactose) [35]. *E. coli *also grows well under anaerobic and aerobic conditions, and can sustain high glycolytic flux. However, ethanol yield is poor because under fermentative conditions *E. coli *also produces lactic, acetic, formic, and succinic acids [56]. Homoethanol fermentation in *E. coli *is hindered by redox imbalance. The pathway to ethanol starts from pyruvate, which is cleaved into acetyl-CoA and formic acid by pyruvate formate lyase (PFL) (Fig. 2). Reduction of acetyl-CoA to ethanol proceeds in two steps through acetaldehyde as intermediate; the multienzyme protein AdhE plays the role of acetaldehyde dehydrogenase and alcohol dehydrogenase, each requiring one NADH [57]. Thus, on a triose basis the pathway from pyruvate to ethanol consumes two NADH, while glycolysis to pyruvate only provides one NADH (in conversion of glyceraledehyde-3-P to 1,3-bisphosphoglycerate). Therefore, ethanol production is balanced by other more oxidized products such as acetic acid (no NADH consumed). To circumvent the redox limitation of the endogenous ethanol pathway, pyruvate decarboxylase (PDC) and alcohol dehydrogenase (ADH) enzymes from *Z. mobilis *were expressed in *E. coli*, via a plasmid bearing an artificial *pet *(production of ethanol) operon containing the *pdc *and *adhB *genes [58]. The transformation conferred homoethanol pathway, with ethanol accounting for 95% of the fermentation products. Also, redox balance is possible in the heterologous pathway because conversion of pyruvate to acetaldehyde and CO_2 _by PDC is nonoxidative, requiring only one NADH for the reduction of acetaldehyde to ethanol.

The *pet *operon was integrated into the *pfl *locus of *E. coli *B to take advantage of its strong, constitutive native promoters. However, the recombinants had low PDC expression, and therefore low ethanol yield [59]. Selection on high chloramphenicol or aldehyde indicator plates resulted in analogous mutants with PDC expression comparable to that in the plasmid-bearing strain and *Z. mobilis*. Further deletion of fumarate reductase (Δ*frdABCD*) reduced succinic acid production by 95%; the resulting strain, KO11, produced ethanol at 100% theoretical yield when grown on glucose or xylose in rich medium [59]. Compared to the parent strain, KO11 exhibits higher maximum growth rate (30% higher) and glycolytic flux (50% higher). This is attributed to higher expression of the xylose catabolic genes, which came to light through global expression analysis using DNA microarrays [60]. Directed evolution of KO11 by increasing ethanol concentration from 35 to 50 g/L resulted in strain LY01, which fermented xylose to 60 g/L ethanol titer with 85% yield [61,62]. Microarray analysis revealed increased glycine metabolism and betaine synthesis in LYO1 compared to KO11, therefore linking ethanol tolerance to osmotic stress (glycine and betaine are protective osmolytes) [63]. Addition of glycine or betaine was shown to increase ethanol tolerance in KO11 and allowed for the fermentation of 9% (w/v) of xylose to 4% (w/v) ethanol in 48 hours [62]. More recently Kim *et al*. reported homoethanol fermentation from xylose and glucose using native *E. coli *genes with yields up to 82%, by combining the activity of pyruvate dehydrogenase, usually aerobic, with the alcohol dehydrogenase one [64].

Arabinose is another pentose sugar obtained upon deconstruction of biomass. There are two different arabinose utilization pathways in nature, bacterial and fungal. The bacterial pathway is redox-balanced and encompasses three enzymatic steps, whereas the fungal pathway consists of five enzymes, including four oxidoreductases, and is characterized by a redox imbalance. Both pathways have been independently expressed in yeast [65,66]. The results obtained with the recombinant *S. cerevisiae *strain engineered with the heterologous fungal pathway showed growth on L-arabinose, although at a very low rate [66]. Recently, a mutant yeast strain which anaerobically converts arabinose to ethanol in batch fermentation was reported [67]. This strain was obtained by introducing the bacterial pathway for arabinose utilization from *Lactobacillus plantarum*, overexpressing *S. cerevisiae *genes encoding the nonoxidative PPP enzymes, and subsequent evolutionary engineering. An ethanol yield of 0.43 g/g carbohydrate consumed and a specific ethanol production rate of 0.29 g/g/h from arabinose as the sole carbon source were achieved.

#### Conversion of sugars to butanol

**Figure 3:**
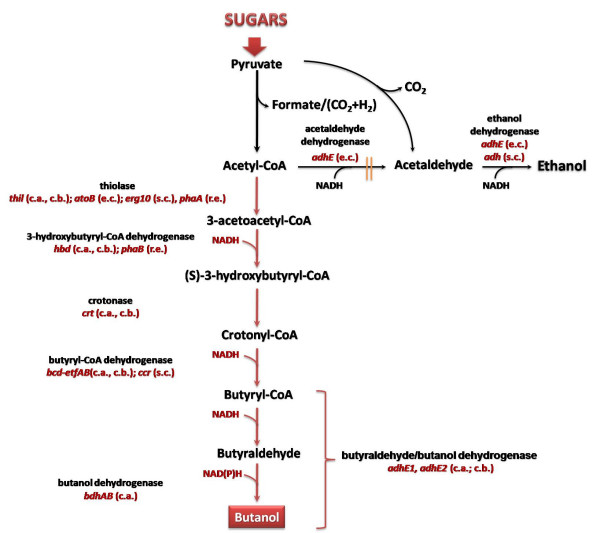
**Current metabolic engineering efforts for 1-butanol production**. Heterologous expression of clostridial genes in *S. cerevisiae *and *E. coli*. Gene names in red indicate steps engineered for butanol biosynthesis. Source of the genes is reported in parenthesis. *c.a*., *Clostridium acetobutylicum*; *c.b*., *Clostridium beijerinckii*; *e.c*., *Escherichia coli*; *s.c*., *Saccharomyces cerevisiae*; *r.e*., *Ralstonia eutropha; s.c*., *Streptomyces collinus*. || indicates gene knock-outs. Gene/enzyme names: *adh*, *adhE1*, *adhE2*, alcohol dehydrogenase;*bcd*-*etfAB*, butyryl-CoA dehydrogenase;*ccr*, butyryl-CoA dehydrogenase;*crt*, crotonase; *erg10*, thiolase; *fdh1*, formate dehydrogenase; *phaA*, thiolase; *phaB*, acetoacetyl-Coa reductase; *thl*, thiolase.

Recently there has been an increased interest to convert sugars from lignocellulosic biomass into butanol. Due to its physical properties, the four-carbon alcohol is a better replacement for gasoline than ethanol [68]. As mentioned above, various clostridia have been utilized in butanol fermentation, although these gram-positive anaerobes coproduce butanol with a few byproducts, such as butyric acid, acetone, ethanol, therefore lowering its yield [69]. From a biotechnology perspective, the lack of efficient genetic tools to manipulate clostridia hinders metabolic engineering endeavors for the optimization of butanol synthesis and the reduction of by-product formation. Because of these reasons, *E. coli *[10,11,70,71] and *S. cerevisiae *[72] were recently engineered for butanol synthesis from sugars [Fig. 3]. The engineering strategy in *E. coli *involved the re-construction of the synthetic CoA-dependent clostridial pathway. Synthetic operons carrying all the necessary genes for bioconversion of Acetyl-CoA to butanol (*thl*, *hbd*, *crt*, *bcd*, *etfAB*, and *adhE2*) were simultaneously expressed in *E. coli *and led to the fermentative production of up to 14 mg/l of butanol from glucose as sole carbon source [70]. This pathway was later optimized by utilization of enzymes from different microorganisms. Replacing the clostridial thiolase gene (*thil*) with the native *E. coli *AtoB (*atoB*) led to divergent results [10,70]. Furthermore, since the clostridial butyryl-CoA dehydrogenase (*bcd*) activity is hypothesized to be a rate limiting step in butanol production [73] and its activity is closely linked to the expression of electron transfer proteins (*etfA*, *etfB*), a heterologous crotonase (*ccr*) from *Streptomyces collinus *was expressed in place of the clostridial one. However, replacing the original enzyme with *ccr *resulted in much lower yields of butanol in *E. coli *[10,11,70]. Since expression of the butanol pathway resulted in low butanol synthesis, some endogenous *E. coli *pathways were disrupted to avoid the flow of carbon to by-products [10,11,70,71]. Combining all of the optimization strategies, the maximum butanol titer and yield in engineered *E. coli *were 1.2 g/l and 6.1 g butanol/g glucose, respectively [71].

A similar synthetic strategy was also investigated in *S. cerevisiae *[72]. The effect of two different thiolases, alternative to the native *S. cerevisiae *Erg 10, on butanol production was evaluated. AtoB, the native *E. coli *enzyme, had been shown to efficiently catalyze the conversion of acetyl-CoA to acetoacetyl-CoA [74], while PhaA from *Ralstonia eutropha *had been previously reported as very active [75] and yielded the highest butanol titer (1 mg/l). Also, since growth under fermentative conditions usually leads to the accumulation of reducing equivalents (NADH), the use of Hbd, a NADH-dependent 3-hydroxybutyryl-CoA dehydrogenase isoform, was investigated as alternative to PhaB, which is NADPH-dependent. While a combination of different isoforms of 3-hydroxybutyryl-CoA with the different thiolases showed that PhaA and PhaB had most likely been optimized by evolution to work perfectly in concert, the best match for butanol production was shown to be the native yeast thiolase (Erg10) in combination with the NADH-dependent 3-hydroxybutyryl-CoA dehydrogenase [72]. Heterologous expression of the crotonase (Ccr) from *S. collinus *in place of the clostridial Etf-dependent led to somewhat higher titers but was still limited to 2.5 mg/l of butanol [72].

**Figure 4:**
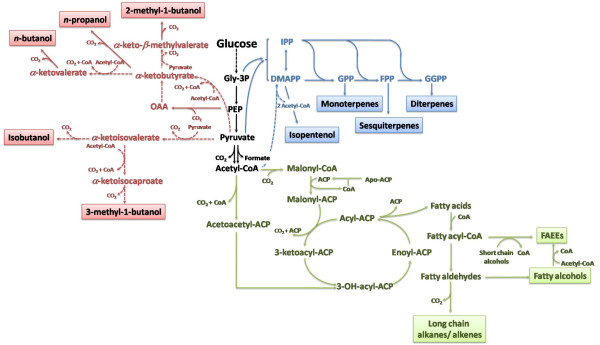
**Synthetic pathways for advanced biofuels**. Fuels generated by the keto-acid mediated pathway are shown in red. Isoprenoids biosynthesis including the mevalonate and methylerythritol pathways is shown in blue. Fatty acid pathway for fatty acids ethyl esters (FAEE), fatty alcohols and long-chain alkanes/alkenes is reported in green. The dashed lines indicate multiple steps. Fuel products are shown in boxes. Abbreviations: ACP, acyl carrier protein; CoA, Coenzyme A; DMAPP, dimethylallyl pyrophosphate; FAEE, fatty acids ethyl esters; FPP, farnesyl diphosphate; Gly-3P, glyceraldehydes-3Phosphate; GPP, geranyl pyrophosphate; GGPP, geranyl-geranyl pyrophosphate; IPP, isopentenyl pyrophosphate; PEP, posphoenolpyruvate.

Butanol production was also attempted in *E. coli *by exploiting the keto-acid mediated pathway, which utilizes norvaline biosynthesis chemistry and the leucine biosynthesis operon (*leuABCD*) [76] (Fig. 4). This strategy was developed to overcome the limitations faced during expression of the synthetic clostridial pathway. It has been indeed hypothesized that low butanol titers achieved in these engineered platforms were most likely due to the oxygen sensitivity and CoA-dependence of the clostridial pathway [76,77]. Implementation of the norvaline biosynthetic pathway and overexpression of its precursor in *E. coli *led to the production of 2-ketovalerate, an intermediate of norvaline biosynthesis, which was channeled to butanol by keto-acid decarboxylase and alcohol dehydrogenase. 2-ketovalerate was produced through the *leuABCD E. coli *pathway from the 2-ketobutyrate generated from L-threonine by the product of the gene *ilvA *[76]. Overexpression of *ilvA-leuABCD *led to a three-fold increase in butanol synthesis. It was also shown that threonine availability was a limiting step, since addition of exogenous threonine led to higher titers. Additionally a titer of 2 g/l with approximately 0.85 g/L of butanol was achieved by deregulating aminoacid biosynthesis and eliminating competing pathways [76]. An alternative route to 2-ketobutyrate synthesis is provided by the citramalate pathway, identified in *Leptospira interrogans *and *Methanocaldococcus jannaschii*; the citramalate synthase converts pyruvate to 2-ketobutyrate in a one step reaction. Directed evolution of this enzyme was used to develop a mutant with higher catalytic activity and insensitivity to isoleucine feedback inhibition, which allowed reaching titers 22-fold higher compared to the native enzyme [78].

Several species of solventogenic bacteria are also very attractive from a biofuel production standpoint because of their capacity to utilize pentose sugars and certain other complex carbohydrates. Solvent production in clostridia is characterized by two physiological phases, acidogenic and solventogenic [79]; the latter, during which acetone, butanol and ethanol are synthesized, is closely coupled to sporulation [80]. Metabolic engineering strategies in clostridia have aimed at improving the selectivity of products synthesis, increasing butanol tolerance and broadening the substrate utilization range [81]. One of the earliest attempts in this direction was the overexpression of the native clostridial pathway for acetone production. Butanol and acetone pathways are indeed partially coupled [79]. Amplification of the genes encoding for the acetoacetate decarboxylase and CoA transferase led to an increase in the amount of precursor of butanol production, butyryl-CoA, and as a consequence to higher butanol titers [82]. Later studies have investigated the effect of *solR*, a putative transcriptional repressor, which negatively regulates the expression of genes associated with solventogenic metabolism [83]. Although further studies are still needed to elucidate the mechanism of regulation, disruption of the *solR *gene led to an increase in butanol synthesis. Solvent tolerance and stress response in clostridia is very complex and it is linked also to expression of chaperones and modulation of fatty acids synthesis [84]. Amplification of the heat shock gene products (groES, groEL) led to an increase in solvent tolerance, probably due to stabilization of the solventogenic enzymes [85]. Lastly, significant efforts are directed toward the development of a cellulolytic capacity in *C. acetobutylicum *that could allow for direct utilization of cellulose. Cellulanase from *C. cellulovorans *[86], *C. cellulolyticum *and *C. thermocellum *have been expressed in *C. acetobutylicum*, but high cellulose activities have yet to be achieved [81].

#### Sugar conversion to advanced biofuels

Hexose conversion to fuels is currently the most established route for biofuels production and has been further developed to produce a wide array of advanced biofuels (Fig. 4). To date, research into gasoline substitutes has focused largely on ethanol. However, biosynthetic and *de novo *pathways could potentially yield molecules that are similar or identical to those currently found in gasoline. These include straight- and branched-chain alkanes from fatty acids and isoprenoid pathways, in addition to higher alcohols and esters (Fig. 4). The wide array of fatty acids of different chain length and degree of saturation that are microbially produced could potentially provide an ideal mixture for a biofuel blend. Biodiesel from microalgal oil has been extensively investigated, but it is a process that still relies on chemical transesterification to produce the fuel molecules [87,88]. In an alternative approach, Kalscheuer *et al*. recently reported an *E. coli *based process for the bacterial conversion of hexoses to microdiesel [89,90]. The biosynthesis of fatty acids in bacteria is a well understood process, which draws from the pool of Acetyl-CoA [91,92]. *E. coli *cells were metabolically engineered by introducing the PDC and ADH genes (*pdc *and *adhB*), respectively, from *Z. mobilis *for abundant ethanol production (see previous section and Fig. 2). The gene *atfA *for an unspecific acyltransferase from *Acinetobacter baylyi *was introduced to esterify ethanol with the acyl moieties of the CoA thioesters of fatty acids. Heterologous expression of these genes in the recombinant *E. coli *resulted in significant fatty acid ethyl ester (FAEE) biosynthesis. Although the FAEE yields obtained were significantly below the requirements of a viable industrial process, the feasibility of the approach was demonstrated in the study.

Isoprenoids have also been considered as potential source of biofuels [6]. *E. coli *and *S. cerevisiae *strains have been developed for the overproduction of isoprenoids, due to their pharmaceutical and nutritional value [93]. The two common precursors of isoprenoids are isoprenyl diphosphate (IPP) and dimethylallyl pyrophosphate (DMAPP), synthesized from glyceraldehyde-3-phosphate and pyruvate via the methylerythritol pathway or from acetyl-Coa through the mevalonate pathway (Fig. 4) [94]. Recently two genes from *Bacillus subtilis *were reported that can convert the IPP precursor to isopentenol [95]. The heterologous expression of a pyrophosphatase from *Bacillus subtilis *in *E. coli *enabled the production of isopentenol [95]. Additionally, farnesol and farnesene were produced in E. coli and S. cerevisiae using a yeast phosphatase and certain plant terpene synthases, respectively [96]. Even if the engineering of these pathways is still at early stage, these terpene molecules are developed as precursors to diesel fuels and could be potential components of next generation jet fuels [4,97].

Isobutanol, an isomer of n-butanol with a higher octane number, was synthesized in E. coli by overexpressing the ilvIHCD operon, so to divert carbon from pyruvate to 2-ketoisovalerate (Fig. 4). To enhance isobutanol synthesis, competing pathways for pyruvate were disrupted and a slight increase in isobutanol production was observed [98]. Additionally, an acetolactate from B. subtilis with higher affinity for pyruvate was overexpressed and pflB disrupted to allow for pyruvate accumulation. The implementation of the abovementioned strategies led to titers of 20 g/l of isobutanol [98]. The aforementioned keto-acid mediated pathway also enables the synthesis of 5-C alcohols, such as 2-methyl-1-butanol and 3-methyl-1-butanol (Fig. 4). As for butanol production, 2-methyl-1-butanol is synthesized from 2-ketobutyrate. Hence, all the strategies discussed earlier for the synthesis of butanol via the keto-acid mediated are also applicable. The leuABCD operon was disrupted to increase product specificity. A combination of these approaches led to titers up to 1.25 g/l of 2-methyl-1-butanol [99]. Similarly, 3-methyl-1-butanol production shares the same strategy used for isobutanol production, with which it competes for the common precursor 2-ketoisovalerate (Fig. 4). Carbon distribution between the two branches still remains a challenge. Leucine-resistant mutants have been utilized to overcome leucine feedback inhibition and pathways for valine and isoleucine formation have been disrupted. A titer of 1.28 g/l of 3-methyl-1-butanol was achieved [100].

#### Efficient utilization of sugar mixtures

Given the high complexity of lignocellulosic hydrolysates, metabolic engineering has been extensively used to develop recombinant strains of traditionally used ethanol producers such as *S. cerevisiae *and *Z. mobilis*, and enteric bacteria such as *E. coli*, that will efficiently ferment mixtures of glucose and xylose, and in some cases, arabinose [101]. When grown on a mixture of sugars such as those obtained from plant biomass, wild-type *E*. *coli *exhibits sequential consumption of them, which is manifested in diauxic growth/diauxie. This is the result of carbon catabolite repression (CCR)[102], a phenomenon in which the presence of a preferred substrate represses the expression of genes required for the metabolism of other substrates. Glucose is the preferred carbon source for *E*. *coli *and many other organisms, hence glucose-induced CCR is well known as the "glucose effect". Due to CCR, the fermentation of xylose, arabinose and other sugars derived from lignocellulosic biomass is delayed and frequently incomplete resulting in lower productivities and product yields (residual sugars are also problematic for downstream processing of products). Therefore, obtaining recombinant strains capable of efficiently fermenting sugar mixtures is a critical step in converting lignocellulosic sugars into valuable products.

CCR can take place by way of different mechanisms, such as permanent repression, transient repression, inducer exclusion, and inducer expulsion [103]. In *E. coli*, CCR s mediated by the combined action of global and operon-specific regulatory mechanisms, referred to here as sugar-utilization regulatory systems (SURS). Global pathways that mediate SURS include the transcriptional activator CRP (cyclic AMP (cAMP) receptor protein), the signal metabolite cAMP, the enzyme adenylate cyclase, and the enzyme IIA component of the glucose-specific phosphoenolpyruvate:carbohydrate phosphotransferase system (PTS) (EIIA^Glc^; also called catabolite repression resistance (Crr) or EIIA^Crr^). The PTS uses phosphoenolpyruvate as an ATP equivalent in active sugar transport. The glucose PTS protein IIAglc (*crr*) also exerts regulatory control on intracellular level of cAMP, which is an allosteric effector required in expression of catabolic enzymes for other sugars [104]. Thus the PTS is responsible for the "glucose effect", i.e., glucose represses the utilization of less preferred carbon sources (e.g., xylose, arabinose), resulting in diauxic growth and sequential consumption of sugars. Disruption of the PTS relieves glucose repression on xylose and arabinose, and hence simultaneous sugar uptake takes place [101,105,106]. Because of impaired transport system, these PTS^- ^mutants would exhibit decreased sugar uptake rates and therefore slower growth, but this deficiency is ameliorated by the activation of an alternative glucose transport and phosphorylation system composed of a glucose facilitator protein (or a galactose permease) and the enzyme glucokinase [107]. This has been achieved in the above examples by either overexpressing the aforementioned components or by using an adaptive evolution approach that selects for PTS^- ^variants that have recovered the ability to grow on glucose. An alternative approach to avoid CCR is the use of CRP mutants that do not require cAMP to activate the expression of catabolic genes (CRP* mutants) [102].

A recent study from Yomano and coworkers showed that disruption of the methylglyoxal synthase gene enhanced co-metabolism of sugar mixtures [108]. Methylglyoxal, an intermediate in the methylglyoxal bypass that diverts carbon from dihydroxyacetone phosphate, is generally regarded as an inhibitor of sugar consumption [109,110]. Deletion of *mgsA *had already previously proven to improve lactate production and was now applied to construction of ethanologenic *E. coli *[108].

### Production of biofuels from non-carbohydrate feedstocks

Bio-oils consist of fatty acids (FAs) bonded to a backbone structure, typically glycerol, and therefore they are generally found in the form of triglycerides (i.e. triesters of FAs and glycerol). Bio-oils are not only abundant, but their use for fuel production also offers several advantages that translate into higher biofuel yield when compared to their production from lignocellulosic sugars. The advantages of using bio-oil components (i.e. glycerol and FAs) for biofuel production, along with recent engineering efforts in this area, are discussed below.

#### Conversion of fatty acids to biofuels

**Figure 5:**
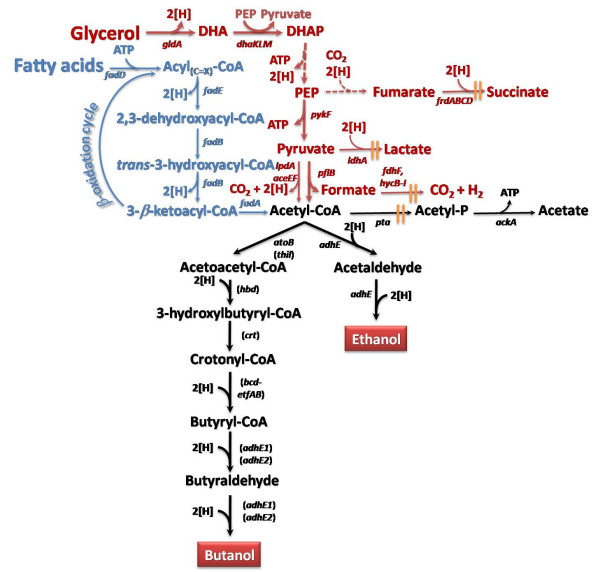
**Engineered pathways for microbial production of biofuels from bio-oil constituents glycerol and fatty acids**. Glycerol dissimilation pathway is shown in red. Fatty acids catabolism is shown in blue. The dashed line indicates multiple steps. || indicates gene knock-outs. Fuels are reported in red boxes. Genes in parenthesis indicate clostridial synthetic steps engineered in *E. coli *for butanol production. 2 [H] = NAD(P)H = FADH_2 _= H_2_. Gene/enzyme names: *adhE*, *adhE1*, *adhE2*, alcohol dehydrogenase; *atoB*, thiolase;*dhaKLM*, dihydroxyacetone kinase; *bcd-etfAB*, butyryl-CoA dehydrogenase; *fadA*, 3-ketoacyl-CoA thiolase; *fadB*, enoyl-CoA hydratase, 3-hydroxybutyryl-CoA epimerase; *fadD*, fatty acyl-CoA synthetase; *fadE*, acyl-CoA dehydrogenase; *fdhF*, formate dehydrogenase; *frdABCD*, fumarate reductase; *gldA*, glycerol dehydrogenase; *hbd*, *β*-hydroxybutyryl-CoA dehydrogenase; *hycB-I*, hydrogenase; *ldhA*, lactate dehydrogenase; *lpdA*-*aceEF*, pyruvate dehydrogenase; *pflB*, pyruvate formate lyase; *pykF*, pyruvate kinase; *thil*, thiolase. Abbreviations: DHA, dihydroxyacetone; DHAP, dihydroxyacetone phosphate; PEP, posphoenol pyruvate.

The metabolism of FAs to the key intermediate metabolite AcCoA is very efficient as it results in 100% carbon recovery (Fig. 5). Since many biofuels can be derived from AcCoA, high yields can be realized in their synthesis from FAs. In contrast, sugar metabolism generates one molecule of carbon dioxide (or formate) per molecule of AcCoA synthesized via glycolysis, severely limiting C-recovery and hence the yield of products derived from AcCoA (Fig. 3 and 4). Another advantage of FAs over sugars is their higher energy content (i.e. higher reduced nature of their carbon atoms), which also results in higher yields of biofuels. Despite these advantages, metabolism of FAs requires the presence of an external electron acceptor, which in turn precluded the synthesis of metabolic products. To overcome this hurdle, our group engineered synthetic respiro-fermentative routes for the efficient production of fuels and chemicals in combination with the effective degradation of FAs (Dellomonaco and Gonzalez, unpublished). *E. coli *was chosen as model organism to illustrate the feasibility of this approach, which was demonstrated by engineering the synthesis of biofuels ethanol and butanol (Dellomonaco and Gonzalez, unpublished). The yield of ethanol in the engineered strain (1.08 g ethanol/g of FAs) was two-fold the maximum theoretical value that can be achieved with the use of lignocellulosic sugars (0.51 g ethanol/g sugar). Butanol, on the other hand, was produced at yields and titers between two- and three-fold higher than those reported for its production from sugars in engineered *E. coli *and *S. cerevisiae *strains [71-73,77].

#### Conversion of glycerol-rich feedstocks to biofuels

Glycerol (or glycerin) is a byproduct of biodiesel, oleo-chemical, and bioethanol production processes. Due to the tremendous growth of the biofuels industry, glycerol is now regarded as a waste product with often a disposal cost associated to it [111]. Given the highly reduced nature of carbon atoms in glycerol, additional advantages can be realized by using glycerol instead of sugars. For example, conversion of glycerol into the glycolytic intermediates phosphoenolpyruvate (PEP) or pyruvate generates twice the amount of reducing equivalents produced by the metabolism of glucose or xylose (Fig. 2 and 5). Fermentative metabolism would then enable higher yield of fuels and reduced chemicals from glycerol compared with those obtained from common sugars such as glucose or xylose.

*E. coli *cannot grow anaerobically due to an imbalance of the redox potential, e.g. accumulation of reducing equivalents [112]. Trinh and coworkers have considered four different electron acceptors to test in silico the anaerobic conversion of glycerol [113]. Electron acceptors can be in the form of substrates added to the medium or pathways that serve as redox sinks. Production of 1,3-propanediol, activation of the methylglyoxal pathway to produce 1,2-propanediol or substrates (e.g. fumarate, nitrate) feeding have been investigated as potential mechanisms enabling glycerol fermentation. Trinh and coworkers utilized oxygen as electron acceptor and employed elementary mode (EM) analysis to design an *E. coli *platform with minimized metabolic functionality that could efficiently convert glycerol to ethanol under microaerobic conditions. *E. coli *was tailored to efficiently produce ethanol from glycerol by inserting nine gene knock-outs (Δ*zwf *Δ*ndh *Δ*scfA *Δ*maeB *Δ*ldhA *Δ*frdA *Δ*poxB *Δ*pta *Δ*mdh*) [113].

Although many microorganisms are able to metabolize glycerol in the presence of external electron acceptors (respiratory metabolism)[112,114], few are able to do so fermentatively (i.e. in the absence of electron acceptors). Fermentative metabolism of glycerol has been studied in great detail in several species of the *Enterobacteriaceae *family, such as *Citrobacter freundii *and *Klebsiella pneumoniae*. However, the potential for using these organisms at industrial level is limited due to their pathogenicity, requirement of strict anaerobic conditions, the need for supplementation with rich nutrients, and a lack of appropriate genetic tools and physiological knowledge necessary for their effective manipulation.

A recent development in the microbial fermentation of glycerol is the discovery by our group that *E. coli*, an organism considered the workhorse of modern biotechnology, can anaerobically ferment glycerol [115-117]. Identification of pathways and environmental conditions affecting the metabolism of glycerol under anaerobic condition by wild type *E. coli *provided opportunities to manipulate this microorganism for enhancement of ethanol yield and productivity, as described below and shown in Figure 5.

Fermentation of glycerol to either ethanol and H_2 _or ethanol [115,118] and formate is one of the most effective ways of exploiting the reduced property of glycerol for the production of biofuels. Disruption of genes that encode fumarate reductase (FRD) and phosphotransacetylase (PTA) in *E. coli *allowed for ethanol-H_2 _production [115]. FRD and PTA are two key enzymes involved in the production of succinate and acetate, respectively [Fig. 5. The resulting strain (SY03) produced almost equimolar amounts of ethanol and hydrogen at a yield comparable to theoretical maximum of 1 mol of each product per mol glycerol [115]. To facilitate co-production of formate and ethanol, an additional mutation was introduced in the gene (*fdhF*) encoding a component of the formate-hydrogen lyase (FHL). FHL is responsible for the oxidation of formate into H_2 _and CO_2_. This triple mutant strain, called SY04, produced exclusively ethanol and formate at yields 92-96% of the theoretical maximum. However, the strategies used in the generation of these mutants led to decrease in the growth rates of *E. coli *mutants [115]. The overexpression of glycerol dehydrogenase (GldA) and dihydroxyacetone kinase (DHAK), responsible for converting glycerol into glycolytic intermediate dihydroxyacetone phosphate, was assessed for improving the growth rate of *E. coli *mutants. Overexpression of GldA and DHAK in the triple mutant SY04 led to production of ethanol and formate at maximum volumetric rates of 3.58 and 3.18 mmoles/L/h, respectively. Similarly, overexpression of GldA and DHAK in SY03 with mutations in two genes, *frdA and pta*, for co-production of ethanol-H_2 _led to production of ethanol at 4.6 mmol/L/h and ethanol and H_2 _yields at 0.96 mol per mol glycerol utilized, respectively. In a more recent study, Hu and coworkers [118] demonstrated higher growth rates along with increased hydrogen and ethanol titers under anaerobic conditions by using adaptive evolution and chemical mutagenesis. The utilization of microaerobic conditions was lately exploited as a means of eliminating the need for rich nutrients [119]. Availability of low amounts of oxygen enabled redox balance while preserving the ability to synthesize reduced products. Experiments involving various mutants confirmed the role of both respiratory and fermentative pathways of glycerol utilization under microaerobic conditions and this metabolic process was harnessed by engineering strains for the efficient co-production of ethanol and hydrogen and ethanol and formate [119].

Lately, production of butanol from glycerol has also received increased attention [10,11,120,121]. In recent years, research in this context has been mainly focused on clostridial species, namely *Clostridium acetobutylicum *and *Clostridium beijerinckii*, for which a pathway for butanol production from glucose has been established [83,122,123]. While *C. acetobutylicum *can metabolize glycerol in the presence of glucose [124], *Clostridium pasteuranium *can grow on glycerol as sole carbon source [120,121]. Butanol production in *C. pasteurianum *on biodiesel-derived crude glycerol has been established [120,121]. Taconi and coworkers reported yields up to 0.36 g butanol/g glycerol [120]. Although this study proves the capability of this strain to grow and produce solvents on crude glycerol, growth of cultures was extremely slow (25 days), limiting therefore its immediate industrial applicability.

As aforementioned, *E. coli *offers a number of advantages compared to clostridia in terms of ease of industrial application and availability of genetic tools. Butanol production in *E. coli *on glucose has been established, and extensively reviewed above; conversion of glycerol to butanol has also been recently reported [10].

### Conclusions and future directions

Metabolic engineering and the most recent synthetic biology have been crucial as enabling technologies for biofuels production, as evident in improvements of biocatalysts and the biomass feedstock itself. In surveying the literature on biocatalyst engineering, recurring themes emerge, namely strategies of the heterologous gene expression, evolutionary selection, and "reverse" metabolic engineering. Advances in the "*omics*" sciences are producing quantum leaps in our knowledge by probing cellular changes associated with new phenotypes and driving the construction of efficient microorganisms for biofuels production.

Latest advances in synthetic biology, metabolic engineering and systems biology will continue to power the development of cell factories producing substantial amounts of biofuels [6-9,11,13,15,16]. Formal integration of systems biology tools such as transcriptomics, proteomics, metabolomics, and fluxomics will support the characterization of new mutants and new metabolic pathways for the production of the desired fuel-grade products, thus contributing to the strain optimization process. The improvement of metabolic models will provide a better description of the physiological behavior of the cells and faster identification of targets for genetic modifications and further metabolic engineering. In addition, the development of detailed kinetic models that include accurate regulatory network parameters will facilitate the identification of enzymatic bottlenecks in the metabolic pathways that could be harnessed in order to achieve biofuels overproduction.

## Competing interests

The authors declare that they have no competing interests.

## Authors' contributions

CD, FF and RG conceived the study. CD performed the literature review and drafted the manuscript. FF and RG provided advice on organizing the manuscript and on editorial quality for all sections. All authors read and approved the final manuscript.
